# The Permanent Removal of Hair by Electrolysis

**Published:** 1882-04

**Authors:** George Henry Fox


### SELECTIONS.

### THE PERMANENT REMOVAL OF HAIR BY ELECTROLYSIS.

The growth of hair upon the female face, to the treatment of which I again invite attention, is a deformity which is very frequently observed. Perhaps few physicians have an adequate idea of its prevalence. In nearly every "museum of living curiosities" a bearded woman figures as one of the chief attractions, and it is quite probable that but a small proportion of bearded women are willing to advertise their misfortune for pecuniary gain. I think there are at least a half-dozen of this class on exhibition throughout the city. Of the number of ladies in private life who endeavor, by articles of various kinds, to conceal the unpleasant fact that they have or might have a beard, it would be very difficult to form an estimate. I have no doubt that there are hundreds of such cases. I speak now merely of those who might raise a thick and long growth of hair which would deserve the name of beard. Of those who have a comparatively moderate growth upon the face, and particularly upon the chin, the number is beyond computation. We note instances of this hypertrichosis on every hand—in the drawing-room, upon the street, or wherever ladies congregate, and, could we but know the secrets of the *boudoir*, we would be surprised to find how large a percentage of our female acquaintances resort occasionally, if not habitually, to the use of the depilatory, the razor, or the tweezers.

Frequently the opinion and advice of the physician are sought respecting this abnormal and obnoxious growth—and what does he say? In all probability he will tell the patient that depilatories are merely palliative, advise her to pull the hairs out or to let them alone, and declare that it is a very trifling matter, and perhaps add jocosely that it is not likely to cause death. Such an opinion never satisfies the patient, for no woman ever yet derived consolation from the fact that ugliness is not fatal. Furthermore, the opinion is not sound. This abnormal growth of hair is not always a trifling matter. It may not kill the patient, it is true; but it is certain to occasion great annoyance. It is very apt to affect her disposition, and to injure her prospects in life, especially if she be young and unmarried; and it may eventually ruin both her health and her happiness, by producing a mental disquietude which in many instances verges on melancholia. The frequent occurrence of facial hairiness among insane women has been observed by several writers, and although in such cases the insanity has usually preceded the abnormal growth of hair, I have no doubt that in many instances the mental worry caused by slight facial hairiness has acted as an exciting cause, and served to develop an insane tendency. Dr. J. C. White mentions the case of a lady who searched long for a surgeon who would flay the lower part of her face, and thus remove the obnoxious growth. I have certainly treated one or two females who were monomaniacs on the subject of their facial hairiness, even when this has been very slight, and the most satisfactory result of treatment in these cases has been the improvement in general health which has followed the removal of the hair which served to make their lives unhappy.

The operation for the permanent removal of hairs by electrolysis has been described by several who have written on the subject, and the descriptions differ merely in a few non-essential points. The operation is a simple one, which any physician with a steady hand and keen eye can readily perform, although, as in many other simple operations, a peculiar dexterity is required, and far more satisfactory results are obtained after a certain amount of experience. An ordinary galvanic battery is required, and a fine needle, which is to be attached to the negative cord. The number of cells required for the operation depends upon the activity of the battery, the delicacy of the patient's skin, and the strength of the hairs to be removed, and should be determined in each case by the effect which is produced. I commonly use from ten to sixteen cells of a zinc-carbon battery, or a corresponding number of a chloride of silver battery.

Upon the style of needle employed depends, in a large measure, the success of the operation. A fine cambric needle, which has been recommended, may be successfully used, but, on account of its stiffness, it is more difficult to introduce it into the follicle without piercing the follicular wall than the hair-like flexible steel broach which I have recommended and invariably use. The cambric needle being larger, is also productive of more inflammatory reaction, and more likely to leave permanent traces of the operation. Formerly I used a very fine platinum wire, pointed by means of a jeweller's file ; but the delicate flexible broach, much finer than those commonly employed by dentists in extracting nerves, is far superior to any other needle which I have ever seen, and is almost a necessity in removing the hairs from the upper lip without the production of a scar. The needle can be readily attached to the end of the battery cord by a few turns of copper wire protected by an inch or more of rubber tubing, or a special handle may be made for the purpose.

Provided with battery and needle, the next thing is to get the patient into a proper chair and in a proper light. A high reclining-chair and a southerly bay-window are desirable, but the main point is to secure sufficient light and to have the operator's eyes upon a level with the patient's chin. The needle is now introduced into the follicle by the side of the hair. If this is skilfully done, no pain whatever is felt by the patient. The sponge-cup or sponge-tipped positive electrode should now be used to complete the circuit. This may be applied to the skin in the immediate vicinity of the hair if but a few cells are used, but it is usually more convenient to allow the patient to hold the positive electrode in one hand, and when the needle has entered the follicle, to ask her to complete the circuit by applying the moistened sponge to the palm of the other hand. The electrolytic action now manifests itself subjectively in the form of a sharp, stinging sensation, and objectively in the form of slight hyperæmia around the needle. In a few seconds the hyperæmia will give place to a blanching of the skin, and a little froth will appear at the mouth of the follicle. If the hair be now seized with a pair of forceps and the gentlest traction exerted, it will be found to be loose in the follicle in the course of from ten to twenty seconds, provided the needle has been skilfully introduced. Before withdrawing the needle the patient should remove her hand from the sponge, in order to avoid the slight shock which would otherwise be felt.

The operation is by no means a pleasant one, but rarely does a patient make any complaint of pain. The majority say it is not as unpleasant as having teeth filled in a dentist's chair, and with the fine needle the painful sensation is greatly reduced. At the first sitting the patient is often nervous, and suffers really more than in a dozen subsequent operations. When the sitting is prolonged, and especially in a poor light, the removal of the hair is very trying to the eyes of the operator. At certain times I know that I have suffered quite as much or even more than the patient. The use of a lens held in the hand or fixed before the eye has been suggested, but for my part I find one of no value. A delicacy of touch and steadiness of hand are more essential in this operation than an unusual keenness of vision.

As to the number of hairs which can be removed at one sitting, I

would say that from thirty to fifty is the number which I usually expect to destroy in an operation lasting three-quarters of an hour. Upon the neck it takes much longer to destroy hairs than upon the chin or cheeks. I have removed over two hundred hairs at one sitting, when patients from a distance were anxious to leave the city; but I deem it far better to spare one's eyes and to be more thorough, even if it involves a greater number of sittings.

If the operation is very skilfully performed, it ought not to leave scars, as a rule. In some cases it is impossible to prevent the production of minute punctate cicatrices, which, however, can be seen only on close inspection. I made a mistake in some of my earlier cases, in operating upon two or more coarse hairs very close together, instead of taking one here and there at short distances apart. A little attention to this hint may serve to prevent the production of slight scarring by those who may attempt the operation. Here again I must refer to the fine needle, for its use greatly lessens the liability to the production of scars.

As regards the immediate success of the operation, it must be stated that, as a rule, a certain percentage of hairs will return and demand removal a second time. I used to expect a return of from thirty to fifty per cent of the hairs, while now I am surprised if from five to ten per cent reappear. In one case, in which I removed over fifty hairs with unusual care, not a single one has returned after an interval of three months. In some patients the growth of hair appears to have ceased, for some unknown cause, and when the hairs are destroyed the cure is effected. In other patients the fine hairs are constantly growing larger and darker, and after the most conspicuous have been removed a new growth will in time succeed, and appear, perhaps, like a return of those previously removed.

In this operation for the permanent removal of hair the question arises as to how the electricity destroys the papilla from which the hair springs. Is it by thermic or by electro-chemical action? A recent writer on the subject objects to the use of the term electrolysis as being a misnomer, claims that the heat generated in the needle by the passage of the electricity is the active agent in the destruction of the tissue, and suggests for the operation the name of *akido-galvano-cautery.* It cannot be denied that in this operation the temperature of the needle is slightly raised by its resistance to the galvanic current, but surely not to such a degree as to produce a caustic effect. On the other hand, it is evident, from the frothing seen at the mouth of the follicle and other effects, that a decomposition of the water and salts contained in the cutaneous tissues is taking place around the needle and causing the escape of bubbles of hydrogen. This is certainly nothing more nor less than electrolysis.

In conclusion, I would like to refer to the cause of facial hirsuties in females, and I shall speak briefly on this point, for I know very little about it. I have wondered and pondered by the half-hour while operating on cases, and endeavored to find some characteristic common to all of my patients, but in vain. Some are in fine physical condition, while others are debilitated. Some are extremely nervous ; some are not so in the slightest degree. Some are stout and others thin. Some are of dark, and others of light complexion. Some are maidens from twenty to fifty years of age ; while of others who are married, some have children and some have none. The somewhat common idea that the growth of a beard in the female is necessarily associated with masculine traits of character is certainly not founded upon fact, for most of my patients have presented the very highest type of feminine refinement. That facial hirsuties is dependent upon a malformation or imperfect development of the reproductive organs, as some have claimed, is, in my opinion, doubtful. Certainly, an intimate relation between these two conditions has not been satisfactorily proven, save in a few exceptional cases.

The relation of facial hairiness in females to derangement of the nervous system is a subject which has already commanded attention, but has not as yet been sufficiently studied. I have already spoken of the depressed mental condition existing in many of my patients, and which I believe to be not merely a result of the disfiguring growth of hair, but a symptom of general nervous disease, upon which the hirsuties in all probability depends. Excessive growth of hair, whether in the male or female, is an aberration of nutrition, and not a sign of excessive vitality. The Samsons of the present day are clean-limbed and usually short-haired specimens of the human race, and in our highest type of feminine health and beauty there is but a moderate growth of hair. The lady in the museum, whose luxuriant tresses trail upon the floor, is rarely, if ever, well-developed, and, like her bearded sister, furnishes unmistakable evidence of perverted nutrition.

An abnormal growth of hair, whether it be in respect to length or location, indicates an abnormal condition of the nervous system. Precisely what this condition may be, and how it may be remedied, I must leave for others to determine.—*Dr. George Henry Fox, in Medical Record.*

